# Transcripts derived from the neocortical enhancer of *Ctnnb1* promote the enhancer-promoter interaction and maintain *Ctnnb1* transcription

**DOI:** 10.1016/j.cellin.2024.100212

**Published:** 2024-10-11

**Authors:** Chen Zhao, Liang Wang, Junbao Wang, Kuan Tian, Xiaojiao Hua, Fangyu Wang, Yan Zhou

**Affiliations:** Department of Neurosurgery, Medical Research Institute, Zhongnan Hospital of Wuhan University, Wuhan University, Wuhan, 430071, China; Frontier Science Center of Immunology and Metabolism, Wuhan University, Wuhan, 430071, China; Medical Ward, Wuhan Hospital of Traditional Chinese Medicine, Wuhan, 430060, China; Department of Neurosurgery, Medical Research Institute, Zhongnan Hospital of Wuhan University, Wuhan University, Wuhan, 430071, China; Frontier Science Center of Immunology and Metabolism, Wuhan University, Wuhan, 430071, China

Dear editor,

Enhancers are *cis*-regulatory sequences, along with *trans*-factors, that spatiotemporally control gene expressions(Jindal & Farley, 2021; Kim & Wysocka, 2023). Many active enhancers can produce transcripts, including bidirectionally transcribed enhancer RNA (eRNA) and unidirectionally transcribed long non-coding RNAs – enhancer-associated lncRNA (eaRNA). While eRNAs are generally nonpolyadenylated, unspliced and unstable, eaRNAs are mostly polyadenylated, spliced and stable(Sartorelli & Lauberth, 2020; Tan, Biasini et al., 2020). Although previous studies indicated that enhancer derived transcripts may actively promote transcription, some believe they are just byproducts of polymerase II at enhancer sites. We previously revealed the presence of an upstream enhancer for *Ctnnb1*, the coding gene for β-Catenin. The enhancer, named as neCtnnb1 (neocortical enhancer of *Ctnnb1*), was found to maintain *Ctnnb1*'s transcription predominantly in developing cerebral cortex (neocortex) of the brain to promote neurogenesis of excitatory neurons in superficial layers(Wang, Wang et al., 2022). It is unknown whether the neCtnnb1 locus could transcribe eRNA or eaRNA, and if so, does it regulate the expression of *Ctnnb1*?

We first analyzed RNA-seq data of developing mouse forebrains deposited in public databases. Strikingly, transcripts were detected downstream of the most conserved region of neCtnnb1, starting at embryonic (E) day 12.5, with peak expression at E14.5, followed by a gradual decline through E16.5 to birth (P0) (Fig. 1A and **1B**). This temporal expression pattern was further validated using quantitative reverse transcriptase polymerase chain reaction (qRT-PCR) in developing forebrain tissues (Fig. S1A). Notably, the transcription activity from neCtnnb1 coincides with, but around one day precedes with the enhancer activity of neCtnnb1. Therefore, the transcripts were named as *eaRNA*^neCtnnb1^. 5′ and 3′ RACE (Rapid Amplification of cDNA Ends) experiments revealed that *eaRNA*^neCtnnb1^ is 835 nucleotides long with three exons (Fig. 1A and **1B**). And because of the 3’ polyadenylation capture method used in regular RNA-seq, *eaRNA*^neCtnnb1^ is polyadenylated. Notably, the sequence of *eaRNA*^neCtnnb1^ was predicted to have a low protein-coding potential score (Fig. S1B) and shows weak ribosome profiling signals relative to *Ctnnb1* (Fig. 1B, bottom). This suggests that *eaRNA*^neCtnnb1^ is less likely to be translated into a functional protein, indicating its potential role as a regulatory RNA rather than a protein-coding RNA. Next, we carried out *in situ* hybridization of *eaRNA*^neCtnnb1^ on coronal sections of embryonic brains, revealing that *eaRNA*^neCtnnb1^ is predominantly enriched at the ventricular zone (VZ) and subventricular zone (SVZ), where most neural progenitor cells reside and exhibit the highest canonical Wnt/β-Catenin signaling activity (Fig. S1C).

**Fig. 1:**
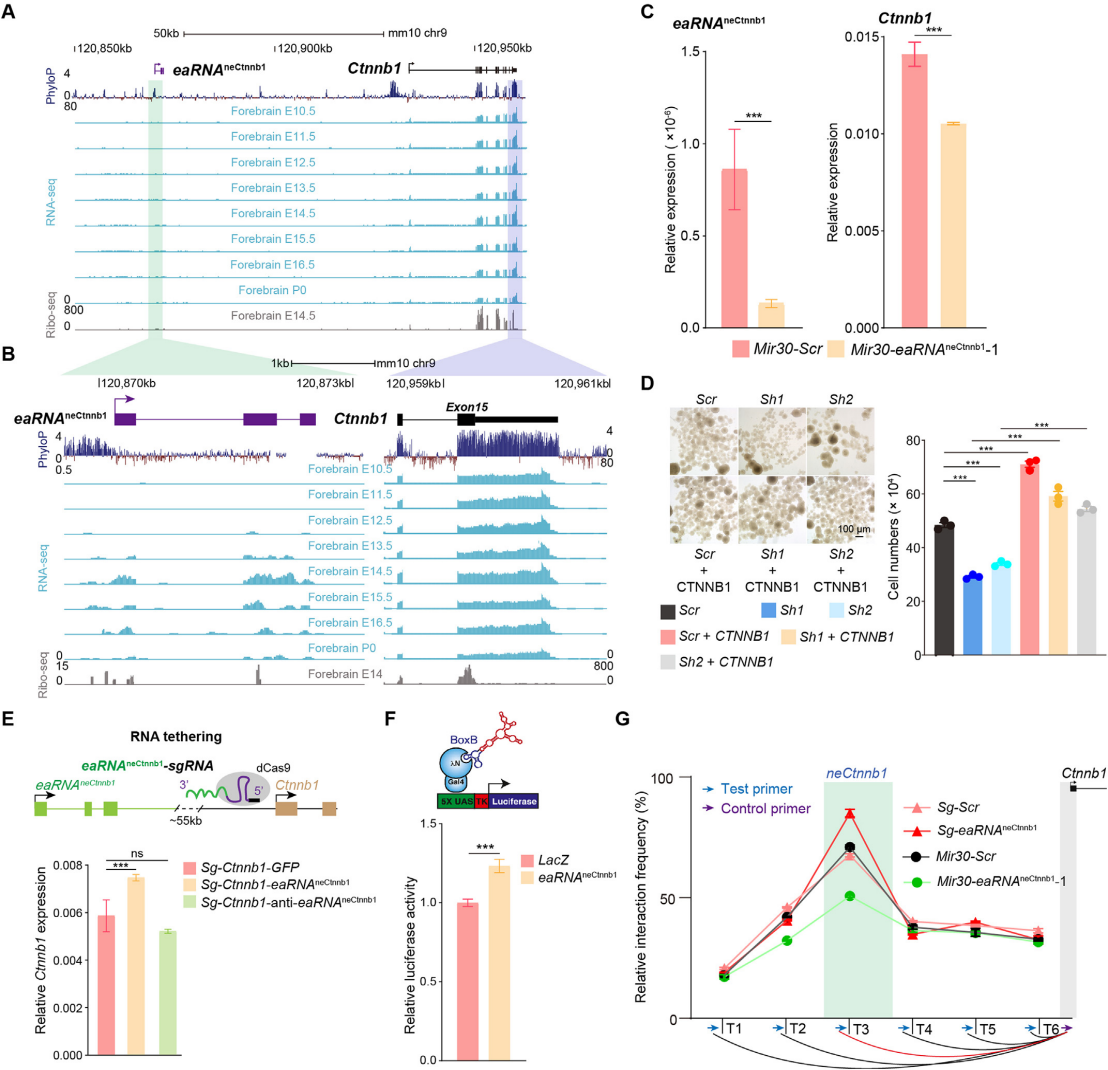
***eaRNA***^**neCtnnb1**^**maintains the transcription of*****Ctnnb1*****and facilitates the interaction of neCtnnb1 and*****pCtnnb1.*** (A–B) Transcripts derived from neCtnnb1 and *Ctnnb1* in forebrains at indicated time points are shown. (B) indicates enlarged regions of neCtnnb1 (green shaded) and the *Ctnnb1* (blue shaded). Please note that *eaRNA*^neCtnnb1^ consists of three exons and is transcribed at the same direction as *Ctnnb1*. The sequence homology among vertebrates is shown at the top, while the Ribo-seq signal of E14.5 forebrains is displayed at the bottom. RNA-seq data were obtained from ENCODE (The Encyclopedia of DNA Elements) and the Ribo-seq data were retrieved from the GEO database (GSE169457). (C) qRT-PCR showing relative mRNA levels of *eaRNA*^neCtnnb1^ and *Ctnnb1* in Neuro-2a cells transfected with shRNAs with scrambled (Scr) sequence or against *eaRNA*^neCtnnb1^ for 72 hours. *n* = 3 independent experiments. (D) Neocortical neurospheres were transfected with lentiviruses expressing indicated shRNAs and human *CTNNB1* for seven days followed by cell counting. *n* = 3 independent experiments. (E) In the RNA-tethering experiments, indicated transcripts were attached with gRNA to target the promoter of *Ctnnb1*. 72 hours after transfection, the expression levels of *Ctnnb1* in Neuro-2a cells were measured by qRT-PCR. *n* = 3 independent experiments. (F) Relative luciferase activity of Neuro-2a cells transfected with plasmids expressing BoxB-tagged *LacZ* or *eaRNA*^neCtnnb1^ along with Gal4-kN and UAS-TK-Luciferase for 24 hours. *n* = 3 independent experiments. (G) In the 3C experiments, the relative association strength between indicated sites with the promoter of *Ctnnb1* were measured in Neuro-2a cells. The test primer T3 is located within the neCtnnb1 region. Quantification data are shown as means ± SEM, statistical significance was determined using one-way ANOVA analysis (D–E) and an unpaired two-tailed Student's *t*-test (C and F). ∗*p* < 0.05, ∗∗*p* < 0.01, ∗∗∗*p* < 0.001, and ∗∗∗∗*p* < 0.0001. ns, not significant.

We next asked whether *eaRNA*^neCtnnb1^ is functional. We devised short hairpin RNAs (shRNAs) against *eaRNA*^neCtnnb1^, which could efficiently downregulate its expression in Neuro-2a neuroblastoma cells. The expression levels of *Ctnnb1* were decreased by ∼30% upon *eaRNA*^neCtnnb1^ shRNA treatment, indicating *eaRNA*^neCtnnb1^ positively regulates *Ctnnb1* transcription (Fig. 1C). Importantly, numbers of cultured neocortical progenitor cells were decreased by ∼40% on loss of *eaRNA*^neCtnnb1^, which could be completely reversed by overexpressing shRNA-resisting human *CTNNB1* (Fig. 1D). Therefore, *eaRNA*^neCtnnb1^ maintains self-renewal of neural progenitors in a *Ctnnb1*-dependent manner. We next explored whether *eaRNA*^neCtnnb1^ has a *trans*-activating role. To this end, the transcript of *eaRNA*^neCtnnb1^ was attached with the guide RNA to target the promoter of *Ctnnb1* (*pCtnnb1*), with the transcript of *Gfp* and antisense *eaRNA*^neCtnnb1^ as controls. Data showed that *eaRNA*^neCtnnb1^ could significantly enhances the transcription of *Ctnnb1* in Neuro-2a cells, whereas the antisense *eaRNA*^neCtnnb1^ was unable to do so (Fig. 1E). Consistently, the Gal4-λN/BoxB reporter assay revealed that *eaRNA*^neCtnnb1^ could boost the luciferase reporter activity (Fig. 1F). Together, *eaRNA*^neCtnnb1^ bears intrinsic activity to promote transcription.

Because *neCtnnb1* physically contacts with the *pCtnnb1*, we then examined whether *eaRNA*^neCtnnb1^ could facilitate the association. Neuro-2a cells were transfected with the shRNA against *eaRNA*^neCtnnb1^. The chromosome conformation capture (3C) assay revealed that downregulating *eaRNA*^neCtnnb1^ could significantly compromise the association of *neCtnnb1* with *pCtnnb1*. Moreover, CRISPR/dCas9-mediated activation (CRISPRa) of the promoter of *eaRNA*^neCtnnb1^ greatly increased the transcription of *eaRNA*^neCtnnb1^ and *Ctnnb1* (Fig. S1D), which simultaneously enhanced the association of *neCtnnb1* and *pCtnnb1* (Fig. 1G). Thus, *eaRNA*^neCtnnb1^ mediates the enhancer-promoter (E-P) contact.

eRNA and eaRNA can help enhancers to find their cognate promoters. Recent research has revealed that repeating sequence within eRNAs and promoter upstream transcripts (PROMPTs) facilitate E-P interactions(Liang, Cao et al., 2023). Here we showed that *eaRNA*^neCtnnb1^, the RNA transcript derived from the neocortical enhancer of *Ctnnb1*, positively regulates the transcription of *Ctnnb1*. *eaRNA*^neCtnnb1^ achieves this possibly by promoting the E-P contact. The *trans*-factor ASH2L has been found to associate with neCtnnb1 and *pCtnnb1*, sustaining *Ctnnb1* transcription(Wang, Wang et al., 2022). It would be interesting to investigate whether *eaRNA*^neCtnnb1^ binds to ASH2L and whether its intrinsic ability to promote transcription depends on this interaction. Intriguingly, a larger proportion of *eaRNA*^neCtnnb1^ is localized in the cytosol than in the nucleus (Fig. S1E), a phenomenon that deserves further investigation. For example, it raises the question of whether *eaRNA*^neCtnnb1^ could regulate the Wnt/β-Catenin signaling by interacting with the post-translational machinery in the cytosol(Lin, Luo et al., 2022). The sequence of neCtnnb1 enhancer is evolutionarily conserved among amniotes with neocortical structures. The possible presence of *eaRNA*^neCtnnb1^ in other species, especially those with complex neocortical structures, might fine-tune the strength of Wnt/β-Catenin signaling, thereby contributing to the expansion of the neocortex during evolution. Recently, we identified another enhancer of *Ctnnb1*, ieCtnnb1 (intestinal enhancer of *Ctnnb1*), which plays a critical role in regulating homeostasis and tumorigenesis of intestinal epithelia(Hua, Zhao et al., 2024). The presence and role of ieCtnnb1-associated RNA also warrant further exploration.

## CRediT authorship contribution statement

**Chen Zhao:** Formal analysis, Data curation, Conceptualization, Investigation, Validation. **Liang Wang:** Formal analysis, Data curation, Conceptualization. **Junbao Wang:** Project administration. **Kuan Tian:** Software, Data curation. **Xiaojiao Hua:** Visualization. **Fangyu Wang:** Funding acquisition, Conceptualization, Project administration, Supervision. **Yan Zhou:** Writing – review & editing, Writing – original draft, Visualization, Validation, Resources, Project administration, Methodology, Funding acquisition, Conceptualization.

## Declaration of competing interest

None.
