# Supplementary material for: Transcripts derived from the neocortical enhancer of Ctnnb1 promote the enhancer-promoter interaction and maintain Ctnnb1 transcription
Source: Cell Insight. 2024 Oct 11;4(1):100212. doi: 10.1016/j.cellin.2024.100212 (PMC11609352; doi:10.1016/j.cellin.2024.100212)
Supplement: Multimedia component 1 [file mmc1.docx]

**Supplementary information for**

**Transcripts derived from the neocortical enhancer of *Ctnnb1* promote the enhancer-promoter interaction and maintain *Ctnnb1* transcription**

Chen Zhao^1,2,#^, Liang Wang^3,#^, Junbao Wang^1,2^, Kuan Tian^1,2^, Xiaojiao Hua^1,2^, Fangyu Wang^1,2,^*, Yan Zhou^1,2,^*

^1^Department of Neurosurgery, Medical Research Institute, Zhongnan Hospital of Wuhan University, Wuhan University, Wuhan, China

^2^Frontier Science Center of Immunology and Metabolism, Wuhan University, Wuhan, China

^3^Medical Ward, Wuhan Hospital of Traditional Chinese Medicine, Wuhan, China.

^#^Equal contribution

*Correspondence: fangyu.wang@whu.edu.cn; [yan.zhou@whu.edu.cn](mailto:yan.zhou@whu.edu.cn)


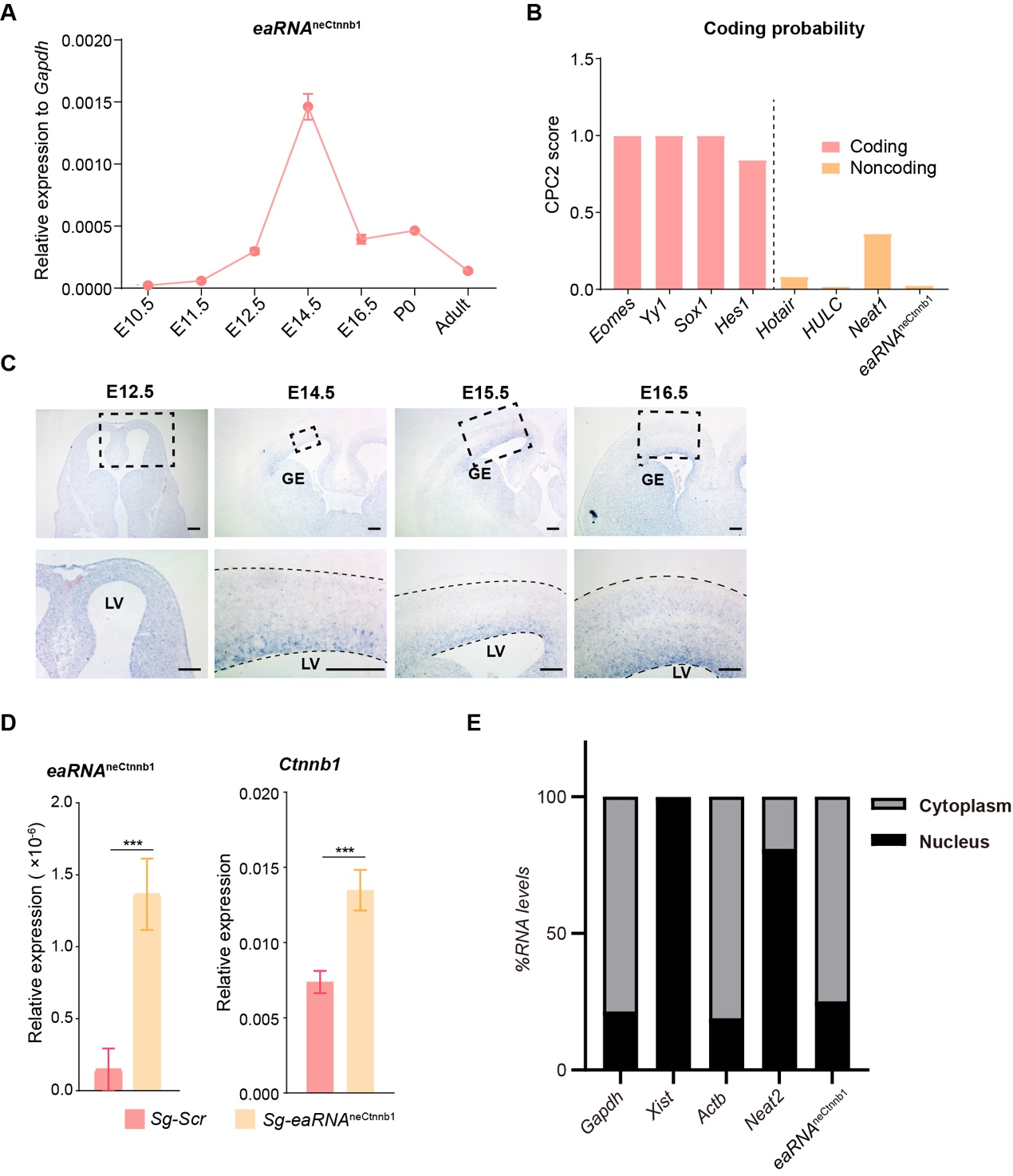


**Supplementary Figure 1.**

(A) Dorsal forebrain tissues at indicated embryonic stages were subjected to qRT-PCR of *eaRNA*^neCtnnb1^. n = 3 embryonic dorsal forebrains. (B) Bar plots showing protein coding potential (CPC2^1^ scores) of indicated protein-coding (left) and non-coding transcripts. (C) Representative images showing *in situ* hybridization signals of *eaRNA*^neCtnnb1^ on coronal sections of forebrain tissues at indicated embryonic stages. Boxed regions on the top were amplified on the bottom. Scale bars, 200 μm. GE, ganglionic eminence; LV, lateral ventricle. (D) RT-qPCR showing relative mRNA levels of *eaRNA*^neCtnnb1^ and *Ctnnb1* in Neuro-2a cells transfected with indicated CRISPR activation vectors for 72 hours. n = 3 independent experiments. (E) Relative expressions of indicated transcripts in cytoplasmic and nucleic fractions of E12.5 dorsal forebrain tissues. *Gapdh*, *Xist*, *Actb*, and *Neat2* are reference RNAs.

**The sequence of *eaRNA*^neCtnnb1^, 835 nt**

GATATTTACTCGGAAAAAGACTGAACGAGCTGAGCATAGCTCCTCCTGCTCTTCCTGGACCTCACAGGCTTGCAGCAGACCCTGCCTGTGCTCAGCCTCTGATGCCTGCCTTGCCTCCCTCTCCCCAGGCCCCAGAAATCAGGAGTGTGAACAAATGTGGAGGGATGAACATGGCAGCAGAAGGTGAAAGGGCTCTGTGGCTGTTGCCCAGCTAACAGTGTGTCTACTGTTGACAGTTTATTCTCATCAAGACAGTGCAGGCTCTGTGGATCCTGTGTGAATCTGTCCATCACCTTGAATGTCTGGCGATCTTGACACCACGCTTCATGGCCAGGCTGGACCCTCTACTAACCGCTGAGGTTTCACTGCTTGCTTCTGACCTCAGAGGACCTCTTCATGCCATCCAGACACCTTCACCTCCATTGCGAATTCCTGATATGCTGATTTTCGTGGTTTCTTCCTCTACCTGTACATCCCTCCACACATCCTACCTCTGATCCAGTGTCTATATACCTACCATCCTTTTGTACAGAGACTCCGGAGTCAGAAATGCTATCAGCTGCCTGTAAAGCTGGTGGGCTTACAGTGTGTGCGTGTGCGTGTGCGTGTGCGTGTGCGTGTGCGTGTGCGTGTGCGTGTGTGTGTGTGTGTGTGTGTAAAGAGATCCTCTGCTTAAGCTCTGCCCAGGGAAGAAAGTCTCTTGGCTGCCTTTGGATCAAGATGTAGAACTCTCTTGGCTCCTCCAGCGCCATGGCTGCCTGGAAGCTGCCATGATGTCAATGGACTGACCCTTTAAACTGTAAGCCAACCCCAATTAAATGTCTTTCTTTATATG

**Oligo sequences**

| **Name** | **Sequence** | **Purpose** |
| --- | --- | --- |
| 5’-RACE-Out | ATGCTATCAGCTGCCTGTAAAGCT | 5’ RACE amplification |
| 5’-RACE-Inner | CATCTTGATCCAAAGGCAGCCAAG |  |
| 3’-RACE-Out | GACAGTGCAGGCTCTGTGGAT | 3’ RACE amplification |
| 3’-RACE-Inner | GAGCCCTTTCACCTTCTGCT |  |
| Sg-*eaRNA*^neCtnnb1^ | GTTCACGTTGTTGCCGGAGC | CRISPR activation |
| Sg-Scr (Scramble) | GGGTCTTCGAGGAAGACCT | RNA tethering |
| Sg-*pCtnnb1* | GTTTCCAATGGAGCGGCGTC |  |
| Sh-Scr (Scramble) | TACAACAGCCACAACGTCTAT | knockdown |
| Sh1-*eaRNA*^neCtnnb1^ | GTCTACTGTTGACAGTTTATT |  |
| Sh2-*eaRNA*^neCtnnb1^ | CTCTGATCCAGTGTCTATATA |  |
| 3C-T1 | CCTGACTGAAGCAGGCATCG | 3C experiments |
| 3C-T2 | GCAGGGAGTTACCCGGTAGAA |  |
| 3C-T3 | AGACTCTTTCTTCCTCTCGTAGGCT |  |
| 3C-T4 | ATGATCAGTGAGAGCCTGAAATGAA |  |
| 3C-T5 | ACTCATCTCTCTCTGCTTCCCAATAT |  |
| 3C-T6 | TGGCACTTCAAGTCTCTTCCAGG |  |
| 3C-Control | GCCACTCCTGGTGTTGGAGG |  |
| *eaRNA*^neCtnnb1^-RT-F | TGTGGCTGTTGCCCAGCTAA | qRT-PCR |
| *eaRNA*^neCtnnb1^-RT-R | AAGCGTGGTGTCAAGATCGC |  |
| \| *Gapdh* qPCR-F \| TCGGTGTGAACGGATTTG \| \| --- \| --- \| | TCGGTGTGAACGGATTTG |  |
| *Gapdh* qPCR-R | GGTCTCGCTCCTGGAAGA |  |
| *Actb* qPCR-F | CTAAGGCCAACCGTGAAAAG |  |
| *Actb* qPCR-R | ACCAGAGGCATACAGGGACA |  |
| \| *Neat2* qPCR-F \| \| --- \| | AGGACAACAGGTGAACGAGC |  |
| *Neat2* qPCR-R | TTCTTGCCACACAGGAAGGC |  |
| *Xist* qPCR-F | GTGCTCCTGCCTCAAGAAGAAG |  |
| *Xist* qPCR-R | CAGAGTAGCGAGGACTTGAAGAG |  |
| ISH-Probe -F | GGGATGAACATGGCAGCAGAAG | *in situ* hybridization |
| ISH-Probe-R | GGTAGGATGTGTGGAGGGATGT |  |

**Material and methods**

**Mice**

All mice studies were performed in accordance with protocols approved by the Animal Care and Ethical Committee at Wuhan University. All mice were in the C57BL/6J background. The noon of the day when the vaginal plug was found was counted as embryo (E) day 0.5.

**5′ and 3′ rapid amplification of cDNA ends (5′ and 3′ RACE)**

Nested primers targeting the *eaRNA*^neCtnnb1^ sequence were synthesized. For 5′ and 3′ RACE, a SMART RACE Kit (Takara Clontech) was used according to the manufacturer’s instructions. The PCR products were cloned into pGEM-Teasy vectors (Promega) and subjected to Sanger sequencing to determine the 5′ and 3′ ends of *eaRNA*^neCtnnb1^.

**Cell culture**

HEK293T cells were gifts from Dr. Hongbing Shu (Wuhan University). Neuro-2a cells were purchased from the Cell Bank of Chinese Academy of Sciences, HEK293T cells were maintained in indicated culture media (DMEM, Gbico) containing 10% fetal bovine serum (FBS) (Life Technologies), Neuro-2a cells were maintained in indicated culture media (MEM, Gbico) containing 10% FBS, non-essential amino acid solution (1×, Gbico, 10370021) and 1mM sodium pyruvate (Gbico, 11360070). Mouse neocortical neural progenitor cells (NPCs) were enriched from E10.5 mouse cortex, cultured on ultra-low-attachment plates (Corning, New York, United States) and maintained in indicated culture media (DMEM/F12, Life Technologies) containing N2 and B27 supplements (1×, Life Technologies), 1 mM Na-pyruvate, 1 mM N-acetyl-L-cysteine (NAC), human recombinant FGF2, and EGF (20 ng/mL each; Life Technologies).

**Lentivirus production and cell infection**

To obtain lentiviral particles, HEK293T cells (5 × 10^6^ cells in a 10-cm dish) were transiently transfected with 12 μg pLKO.1 shRNA constructs, 6 μg of psPAX2 and 6 μg pMD2.G. The supernatant containing lentivirus particles was harvested at 48 hours after transfection and filtered through Millex-GP Filter Unit (0.22 μm pore size, Millipore). Viral particles were then stored at -80°C ultra-cold freezer until use. Knockdown efficiency was evaluated by RT-qPCR analysis three days post-infection.

**CRISPR/dCas9-mediated transcription activation (CRISPRa) assay**

Clustered regularly interspaced short palindromic repeat activation (CRISPRa), in which a nuclease-null Cas9 (dCas9) is fused to transcriptional activators like VP64 domain, enables efficient increase in target endogenous gene expression. CRISPRa assays were performed according to published procedures ^2-4^. SgRNAs were designed to target *neCtnnb1* by using online tool (<https://zlab.bio/guide-design-resources>). SgRNAs were cloned into sgRNA (MS2) cloning vector (Addgene, #61424). For CRISPRa assays, vectors of sgRNA, MS2-P65-HSF1_GFP (Addgene, #61423) and dCAS9-VP64_GFP (Addgene, #61422) were transfected into cells. 48 hours after transfection, cells were collected to extract RNAs and the expression of *Ctnnb1* was quantified by RT-qPCR.

**RNA tethering**

The sgRNA targeting the promoter of *Ctnnb1* was cloned into the pcDNA3.1 vector and tagged with *Gfp*, antisense *eaRNA*^neCtnnb1^ or *eaRNA*^neCtnnb1^ sequences. Neuro-2a cells were transfected with mixed vectors that expressed dCas9 along with sgRNA tagged with *Gfp*, antisense *eaRNA*^neCtnnb1^, or *eaRNA*^neCtnnb1^. Transfected cells were harvested to extract RNAs for assessing *Ctnnb1* RNA levels by RT-qPCR at 48 h post-transfection.

**BoxB-Gal4-λN Luciferase reporter assay**

For BoxB-Gal4-λN luciferase reporter assays, Neuro-2a cells were seeded in 24-well plates for 24 hours and transfected with mixed vectors of 50 ng 5×UAS-TK-Luc, 50 ng pcDNA3-Gal4-λN; and 50 ng pcDNA3-BoxB-*LacZ*/*eaRNA*^neCtnnb1^ along with 5 ng pTK-Ren vectors. Cells were harvested and delivered to the reporter activity testing assay by a Dual-Glo luciferase Assay System on the GloMax Luminometer System (Promega) at 24 hours post-transfection. Data were gathered by calculating the Luc/Ren ratio of triplicate wells.

***In situ* hybridization (ISH)**

Templates for antisense RNA probes were cloned from mouse cDNA with specific primers for *eaRNA*^neCtnnb1^. Then, templates were cloned into the pGEM-T Easy vector (Promega). Vector with target template was linearized by appropriate restriction enzyme and then transcribed to DIG-labeled probes by using the DIG-RNA Labeling Mix (Roche). Cryosections were dried in a hybridization oven for 15 min at 50°C, followed by fixing with 4% PFA for 20 min at room temperature. Then, sections were permeabilized with 2 μg/mL proteinase K (Sigma) in PBS for 10 min at room temperature and acetylation in 0.1 M TEA (triethanolamine) for 10 min at room temperature. After permeabilization and acetylation, sections were blocked in hybridization buffer (5× SSC, 5× Denharts; 500 μg/mL herring sperm DNA; and 250 μg/mL yeast RNA; 50% deionized formamide) for 3 hours at room temperature followed by incubating with a DIG-labeled probe diluted (0.2 ng/μl) in hybridization buffer overnight at 65°C in a hybridization oven. Sections were washed with 0.1 × SSC for four times (20 min each) in a hybridization oven at 65°C, followed by treating with ribonuclease A (TAKARA) (20 μg/ml) for 20 min at 37°C and then blocked with 10% normal sheep serum in Buffer B1 (0.1 M Tris-HCl, pH 7.4; 150 mM NaCl) for 3.5 hours at room temperature. Sections were incubated with 1:5000 dilution of anti-DIG antibody (Roche) overnight at 4 °C. The next day, sections were washed with Buffer B3 (0.1 M Tris-HCl; 0.1 M NaCl; 50 mM MgCl2; 0.1% Tween-20, pH 9.5) for three times (10 min each) at room temperature, followed by colorization with BCIP/NBT (bromochloroindolyl phosphate/nitro blue tetrazolium) (Roche) containing B3 solutions at room temperature in dark. The colorization time depends on the strength of target genes expression. Sections were dehydrated with gradient ethanol and xylene sequentially, and mounted with neutral balsam.

**Cytosol-nucleus fractionation**

Cytosol-nucleus fractionation was performed as previously described^5^. In brief, E12.5 mouse neocortex tissues were dissociated by treating with papain, counted, and centrifuged at 168 ×*g* for 5 min. The pellet was lysed with 175 μL/10^6^ cells of cold RLN1 solution [50 mmol/L Tris-HCl, pH 8.0; 140 mmol/L NaCl; 1.5 mmol/L MgCl_2_; 0.5% NP-40; 2 mmol/L vanadyl ribonucleoside complex (Sangon Biotech)] for 5 min. The suspension was centrifuged at 4 °C and 300 ×*g* for 2 min. The supernatant, corresponding to the cytoplasmic fraction, was transferred into a new tube and stored on ice. The pellet containing nuclei was corresponding to nuclear fractions.

**RNA isolation, cDNA synthesis and quantitative RT–PCR (qPCR)**

Total RNAs were prepared using the RNAiso Plus (TAKARA) according to the manufacturer’s protocols. Tissues or cells were lysed with 1 mL or 500 μL RNAiso Plus (Takara) in DNase/RNase-free EP tubes on ice, followed by adding 200 μL or 100 μL chloroform to achieve phase separation. After shaking vigorously, tubes were centrifuged at 12,000rpm for 15 min at 4°C, and then transfer aqueous phase to new tubes. The aqueous phase was mixed with equal volumes of isopropyl alcohol to obtain RNA. Precipitation was resuspended with an appropriate DNase/RNase-free water. Complementary DNAs (cDNAs) were synthesized by HiScript® II Q RT SuperMix for qPCR kit (Vazyme; R222-01). qPCR primers were designed by PrimerBank ^6^. cDNAs were used to detect different genes with 2× SYBR Green qPCR master mix (Bimake). Amplifications were performed using the CFX Connect Real-Time PCR Detection System (Bio-Rad) with a final volume of 10 μL under the following condition: 5 min at 95°C and then 40 cycles at 95°C for 15 s and 60°C for 20 s. Relative expression levels for target genes were calculated using the 2^−ΔΔCt^ method ^7^, normalized to the *Gapdh* or *ACTIN* housekeeping gene.

**Chromosome Conformation Capture (3C)**

The 3C assay was performed according to the published protocol^8^. In short, Neuro-2a cells were harvested (1×10^7^ cells) and crosslinked with 2% formaldehyde for 10 min at room temperature followed by quenching with 0.125 M glycine for 5 min. Then extracted nuclei were digested by BamH I (NEB Biolabs) overnight at 37°C, followed by treatment with T4 ligase (Takara) for 30 min at room temperature. DNAs were purified by phenol-chloroform extraction and analyzed by qPCR. The specificity and efficiency of all 3C primers were agarose-gel-verified by digestion and ligation of the BAC DNA (RP23-454K9) that contained the region of interest. For each primer pair (test primer to constant primer), we performed a triplicate quantification (Ct1, Ct2, and Ct3), and then calculated the mean Ct. The crosslinking value was calculated using parameters of the standard curve of BAC DNA: value = 10^(Ct-^*^b^*^)^/*a* (*b*: intercept; *a*: slope). These values were finally normalized to *Gapdh* to generate relative crosslinking frequency.

**References**

1 Kang, Y.-J. *et al.* CPC2: a fast and accurate coding potential calculator based on sequence intrinsic features. *Nucleic Acids Research* **45**, W12-W16, doi:10.1093/nar/gkx428 (2017).

2 Alerasool, N., Segal, D., Lee, H. & Taipale, M. An efficient KRAB domain for CRISPRi applications in human cells. *Nat Methods* **17**, 1093-1096, doi:10.1038/s41592-020-0966-x (2020).

3 Konermann, S. *et al.* Genome-scale transcriptional activation by an engineered CRISPR-Cas9 complex. *Nature* **517**, 583-588, doi:10.1038/nature14136 (2015).

4 Wang, J. *et al.* A Ctnnb1 enhancer regulates neocortical neurogenesis by controlling the abundance of intermediate progenitors. *Cell Discovery* **8**, 74, doi:10.1038/s41421-022-00421-2 (2022).

5 Wang, A. *et al.* An epigenetic circuit controls neurogenic programs during neocortex development. *Development* **148**, doi:10.1242/dev.199772 (2021).

6 Wang, X., Spandidos, A., Wang, H. & Seed, B. PrimerBank: a PCR primer database for quantitative gene expression analysis, 2012 update. *Nucleic Acids Res* **40**, D1144-1149, doi:10.1093/nar/gkr1013 (2012).

7 Schmittgen, T. D. & Livak, K. J. Analyzing real-time PCR data by the comparative C(T) method. *Nat Protoc* **3**, 1101-1108, doi:10.1038/nprot.2008.73 (2008).

8 Hagège, H. *et al.* Quantitative analysis of chromosome conformation capture assays (3C-qPCR). *Nature Protocols* **2**, 1722, doi:10.1038/nprot.2007.243 (2007).
